# A Comparative Evaluation of the Apically Extruded Debris from Root Canals Prepared by R-Motion NiTi File System

**DOI:** 10.1155/2023/5731248

**Published:** 2023-04-26

**Authors:** Farah B. Al-Saffar, Hikmet A. Al-Gharrawi

**Affiliations:** Department of Conservative Dentistry, College of Dentistry, Mustansiriyah University, Baghdad 10001, Iraq

## Abstract

**Background:**

Apically extruded debris (AED) is an inherent concern during root canal treatment for both endodontists and general practitioners. The present study investigates the AED of the novel R-Motion single-file reciprocating system compared to standard single reciprocating and multifile rotary systems.

**Materials and Methods:**

Fifty-six moderately curved palatal roots of upper maxillary first molars were selected for the present study. The samples were then divided randomly into four groups (*n* = 14)— Group I: R-motion (RM), Group II: WaveOne Gold (WOG), Group III: ProTaper Next (PTN), and Group IV: HyFlex EDM (HFEDM). The researcher has modified Myers and Montgomery's method to simulate human body temperature. Vials were used to collect debris and weighted using a 0.00001 sensitive balance before and after instrumentation. The instrumentation of all experimented groups was done at 37°C, terminated at master apical file #25. An auto syringe with a side vented needle was used to deliver 8 ml of deionized water for irrigation of each sample during preparation. Vials were stored in a dry sealed desiccator which contained CaSO_4_ crystals, for 24 hr before weighing. The weight of the collected debris was obtained by subtracting the preinstrumentation weight from the postinstrumentation weight for each vial. The Kruskal–Wallis and Mann–Whitney *U* tests were performed to analyse the statistical difference in the amount of debris between the tested groups at a 0.05 significance level.

**Results:**

The RM system produced less debris extrusion than all tested groups, with a significant difference between the former and the WOG and the PTN systems. However, WOG, PTN, and HFEDM showed no statistically significant difference in the amount of AED.

**Conclusion:**

All tested groups produced apical debris in different amounts. The RM produced substantially less AED than WOG and PTN. Meanwhile, WOG, PTN, and HFEDM caused a comparable amount of AED.

## 1. Introduction

The apically extruded debris (AED) during root canal shaping and cleaning is a principal reason for the failure of the endodontic treatment procedure [1]. The chemomechanical disinfection of root canals is defined by shaping the root canals to be appropriately irrigated [2]. During instrumentation, infected pulp tissue remnants, dentinal chips, bacterial byproducts, and irrigation solutions can be pushed throughout the apical foramen to the periapical tissue [3]. Disrupting the integrity of the periapical tissue triggers an immunological reaction that leads to postoperative complications such as flare-ups, which in turn influence the prognosis of the endodontic treatment [4].

The incidence of apical extrusion of debris is multifactorial, and it can be attributed to tooth-related factors such as the type of tooth, root curvature, apical foramen size, and patient age. Moreover, instruments significantly influence AED, including instrumentation techniques, instrument design, alloy, motion, and the number of files under use. Furthermore, irrigation delivery systems and quantity have been identified to play a significant role in expelling root canal material into the periapical tissue [5].

Reciprocation motion was first demonstrated by Yared [6] to improve the mechanical properties of NiTi endodontics files, which later led to the introduction of the single file concept. Meanwhile, NiTi endodontic shaping systems have made numerous enhancements in instrument design and manufacturing techniques since being first classified in 2013 based on their design, metallurgy, and motion [7]. As a result, this concept has become attractive as it saves the clinician's time and reduces patient cross contamination [8]. However, laboratory-based studies have suggested that reciprocation motion may enhance debris transportation toward the apex [9].

Since evidence-based laboratory studies and clinical data showed contradictory results, the debris extruded by this kinematics compared with continuous rotation has caused substantial disagreement. For instance, studies have concluded that a single file reciprocating system caused less debris extrusion than the multifile rotary system [10, 11].

Among the newly advertized reciprocating single-file systems is the R-motion (RM) by (FKG Dentaire, La Chaux-de-Fonds, Switzerland). Its design demonstrates a spherical tip, a rounded triangular cross section combined with a slender core to allow the instrument to effectively cut the canal walls while providing more space for the file to travel along the variable canal anatomy [12].

New advances in endodontic instrumentation attempted to improve instrument properties [7]. AED has been identified as a critical clinical parameter for assessing the efficacy of instrumentation techniques and currently launched instruments [13]. Consequently, many researchers are constantly investigating novel characteristics to provide evidence of their effectiveness [5].

According to a thorough literature review, there are no studies considering the amount of AED using the RM reciprocating single file system compared to either standard reciprocating or multifile continuous rotational systems. Therefore, the present in vitro study aimed to evaluate the amount of AED produced by the RM (FKG Dentaire, Switzerland) compared to WaveOne Gold (WOG) (Dentsply Sirona, Switzerland), ProTaper Next (PTN) (Dentsply Maillefer, Switzerland), and HyFlex EDM (HFEDM) (Coltene, Germany).

## 2. Materials and Methods

### 2.1. Sample Selection

The Mustansririyah University Ethics Committee approved this research before the sample collection began (Reference no.: REC116 15/April/2022). Fifty-six freshly extracted human maxillary first molars within 6 months of extraction were collected from patients ranging in age (from 40 to 60 years) [14]. Extracted teeth were cleaned from the remnants using a periodontal curette, immersed in sodium hypochlorite 5.23% (AQUA, Turkey) for 30 min, rinsed with tap water, and stored in 4°C deionized water.

The criteria for sample selection in this study were mature apex, initial file #15, patent canal, and devoid of any resorptions, cracks, or fractures [15]. First, samples were examined under a digital microscope (Koleertron, China) at 15x to exclude samples that showed cracks or other formerly mentioned defects. Then, the operator recorded radiographic images (Takara, Belmont, Japan), and the image analysis was done by a research assistant using image analysis software (IC Measure 2021, Germany) to eliminate bias. Following radiographic verification, palatal roots with a moderate curve ranging from 0° to 20° according to the Schneider method were chosen in this study [16].

The coronal part of the sample was sectioned to obtain a uniform length of 12 mm. During the decoronation step, the dental lab electric motor (Marathon, Korea) with a diamond disk bur (Komet Dental, Germany) was operated intermittently under water cooling to avoid overheating the roots; refrigerated water was delivered via a plastic syringe. Pulpal tissues of all roots were removed with a barbed broach #10 (Komet Dental, Poland). Then, each canal's apical patency and working length were estimated by inserting a K-file #10 (Komet Dental, Poland) inside the canal and progressing until it was visualized at the apical foramen using magnifying loops (3.5x–420 mm, Zumax, China). Meanwhile, the rubber stopper was leveled at the decoronated root edge as a reference point, the distance was measured with an endodontic ruler, and the working length was calculated by subtracting 1 mm away from the apical foramen.

Finally, the initial file size of each canal was verified using K-file #15 to determine the first file to bind at the full working length. This procedure was done while carefully inserting the file with a slight watch-winding action to avoid forcing the instrument into the apex. The root was excluded from this study when binding was not felt with K-file #15 and the instrument extruded beyond the canal.

Roots were randomly distributed into four groups, each with 14 samples. The sample number was calculated using GPower 3.1 statistical software with 80% power of study (Heinrich-Heine-Universität, Germany) [17].

### 2.2. Instrumentation

Average body temperature was simulated using a digital magnetic hot plate stirrer (Four E's Scientific, China). The device temperature was set by the operator at 37°C, and instrumentation was carried out once the noncontact infrared thermometer (Babyly, China) confirmed a root temperature ranging from 35.5 to 37°C [18].

Eighteeth endomotor (eighteeth E-Connect, China) was used during the instrumentation of roots in all groups according to the instruction of each file system. After establishing canal patency using K-file #10, K-file #15 was utilized as a manual glid path for all samples [19]. The engine was not operated until the file was inserted inside the canal, and when resistance was detected, the file was withdrawn ≈ 1 mm from the binding point. During instrumentation, each tested file was used to prepare three canals only and then discarded. After each file removal, a gauze soaked in 70% ethyl alcohol was used to clean the flutes.

Each canal was irrigated with a total amount of 8 ml of deionized water [20]. Standardizing hand pressure during irrigation was done using an autosyringe (Vista^TM^, USA) with a #30 sideport Endo-Irrigation needle (UDG, Germany) [21]. The flow rate was set at 2.6 ml/min for all samples; the canisters of the autosyringe were filled with the total amount of irrigation required for each sample separately. During irrigation, the needle tip was placed passively into the canal with 2 mm less than the estimated working length to eliminate any chance of the needle binding into the canal walls and to allow backflow of the irrigation solution coronally. Two ml of deionized water was used for irrigation after each file entry of all tested instruments. Following irrigation, all canals were recapitulated using K-file #10.

#### 2.2.1. Group I: RM

The RM #025/06 file was used in reciprocating angles of 150/30 (CCW > CW) [12]. This file was used of 3 mm amplitude while applying minimal apical pressure, and it was removed after three pecking motions in three passes till it reached the entire working length.

#### 2.2.2. Group II: WOG

The Primary 025/07 WOG file was used with reverse reciprocation of 150/30 (CCW > CW) angles [8]. It was used in three pecking motions of 3 mm amplitude while applying minimal apical pressure until it reached the entire working length in three passes.

#### 2.2.3. Group III: PTN

The endomotor was set to rotate at a constant speed (300 rpm) and torque (2 N/cm) [22]. Instrumentation of canals with this system was done using a brushing motion with gentle apical pressure. The operator started this system with an X1 file (017/04) used in one pass, reaching the estimated working length. After irrigation and recapitulation, the X2 file (025/06) was used similarly in two passes until it reached the full working length.

#### 2.2.4. Group IѴ: HFEDM

The HFEDM file size 020/05 was used in one pass with a gentle apical pecking motion, reaching the full working length. Then, the OneFile size (25/∼) was used similarly in two passes until it reached the working length. This system was operated at a speed of 400 rpm and a torque of up to 2.5 cm [23].

### 2.3. Collection of Debris and Storage of Vials

The original methodology of Myers and Montgomery was modified by incorporating a digital magnetic hot plate stirrer to ensure working at 37°C. Empty vials (Brawn, India) were weighted (without rubber stoppers) six times using a sensitive electronic balance with an accuracy of 0.00001 g (Kern-ABT 100-5 M, Germany). Balance calibration was made before the weighing process to minimize errors. The unused vials were stored inside the desiccator until they were utilized. Then, the separated rubber stoppers were punctured using a rubber dam puncture so that roots could be fitted inside the rubber stopper at their coronal 1 mm and secured back on the vials. A laboratory beaker held the root/vial assembly to prevent contamination of the glass vials with fingerprints that would produce inaccurate measurements. Moreover, it contained water of up to 15 ml to raise the root/vial assembly's temperature to 37°C [18]. Further, a rubber dam material was used to cover the top surface of the beaker to isolate the assembly while obscuring vision during instrumentation, and it was ligated with dental floss to ensure fixation. Needle #25 was inserted alongside the rubber stopper to stabilize the pressure inside and outside the vial [24].

**Figure 1:**
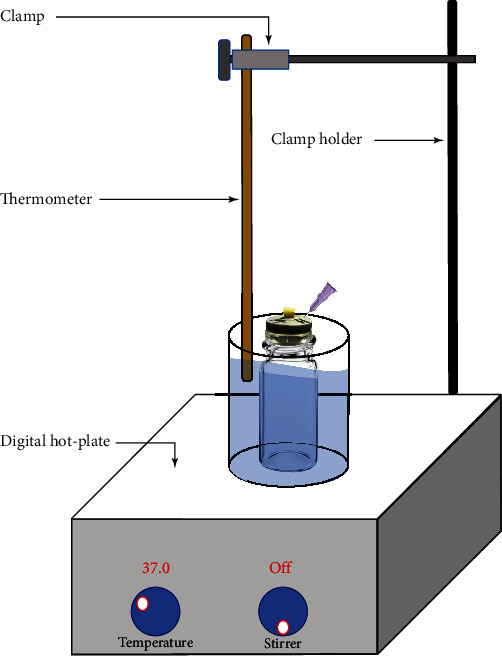
Illustration of the assembly fixed over the magnetic hot-plate stirrer.

Furthermore, the beaker was held in place by a three-finger, vinyl coated adjustable extension clamp (Eisco labs, USA) and fixed by a stainless steel rod. All were then secured to the peripheral rod of the digital magnetic hot plate stirrer (Figure 1).

After the termination of the instrumentation phase, the vial was taken out of the beaker. Then the rubber stopper with the root was removed from the collecting vial, and the root apex was washed off with 1 ml of deionized water using a disposable plastic syringe [25]. The collecting vials were placed in a dry heat oven at 110°C for 3 hr to ensure the dryness of any remaining solution [26]. Then the vials were transported from the oven using Cheatle Forceps and kept in a dry sealed desiccator that contained CaSO_4_ crystals for 24 hr before weighing. After that, vials containing debris were weighed daily until six consistent values were within 0.00001 g [27].

The weight of AED was calculated by subtracting the average preinstrumentation weight from the average postinstrumentation weight for each vial. The resulting difference was considered the weight of the extruded debris.

### 2.4. Statistical Analysis

IBM SPSS Statistics 26 was used to analyze debris weight data (SPSS, Chicago, IL, USA). The Shapiro–Wilk test revealed a nonnormal distribution. Thus, the Kruskal–Wallis test was used to determine the presence of a significant difference between the groups. Mann–Whitney *U* test was performed for comparison between every two groups. The statistical significance value was set at 0.05; greater *P*-values were assumed nonsignificant. Meanwhile, *P*-values equal to or less than 0.05 were considered significant.

## 3. Results

**Figure 2:**
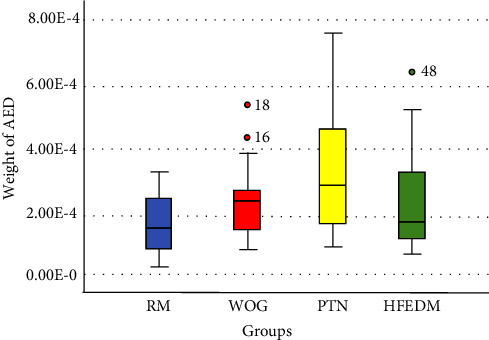
A box plot illustrating the median, minimal, maximal, and standard deviation data of apically extruded debris in all tested groups. A mild outlier is indicated by the (°) circle signal (1.5 as a multiplier interquartile range).

The lowest mean of AED belonged to the RM group, followed by the HFEDM group, while the PTN group had the highest mean of AED, as shown in (Table 1). The Kruskal–Wallis test revealed a significant difference among the test groups. Further, the Mann–Whitney *U* test showed that the RM system extruded significantly less AED than WOG and PTN (*P* < 0.05), However, it produced an equivalent amount to the HFEDM system (Table 2). Likewise, no significant difference between the WOG, PTN, and HFEDM (*P* > 0.05), as illustrated in (Figure 2).

## 4. Discussion

Choosing a root canal shaping system that extrudes the least amount of apical debris improves the endodontic treatment success rate [22]. Thus, many studies have emphasized investigating this parameter when evaluating the clinical performance of newly developed endodontic instrumentation systems [13].

A previous study has reported that postoperative endodontic pain was significantly higher in molar teeth as people advanced in age [28]. Hence, moderately curved palatal roots of the upper first molars were selected from patients ages ranging from 45 to 65 years to correlate the results of this study with the flare-up incidence under a clinical situation.

Standardization of the size of the apical foramen, curvature, and length are strictly followed in this study, as previous study have concluded that larger apical foramina tend to extrude more debris [14]. Thus, the operator in the present study excluded roots with an apical diameter larger than #15.

The adoption of the Myers and Montgomery Methodology in the present study was for its main advantage of assessing the exact quantity of extruded debris with feasibility using a high-precision electronic balance and the possibility of incorporating other modifications to simulate clinical circumstances [13].

The different phase transformation temperatures and composition of various NiTi endodontic instruments resulted in distinct intracanal file behavior [28]. Moreover, to ensure a complete phase transformation (martensite to austenite) of the RM files, which occurs between 32 and 35°C [12], a digital magnetic hot plate stirrer was used in the current study to mimic average body temperature conditions. Meanwhile, the root temperature was confirmed using a noncontact infrared thermometer [29]. Mimicking the periapical tissue resistance could have been done using floral foam. However, it would affect the accuracy of the results by absorbing irrigation solution and debris [13, 17]. Therefore, the periapical tissue was not simulated in the present study.

Careful handling of the sensitive electronic balance was considered in the current study to eliminate the influence of external factors that might compromise the weight conduction procedure [13].

Although using sodium hypochlorite as an irrigation solution would provide a better simulation of clinical conditions, the crystallized solution after the drying procedure would compromise the precise conduction of postinstrumentation weight [13]. For this reason, the operator used deionized water as an irrigant in the present study. Another aspect regarding standardizing irrigation pressure, insertion depth, and volume have been considered in the present study using autosyringa with a side-vented needle gauge 30. The insertion depth was equalized among all samples being 2 mm away from the apex while avoiding any binding during irrigation. A volume of 8 ml of deionized water was used to irrigate each sample to minimize the effect of volume on AED [17]. A single operator did all the steps mentioned in the current study to control operator-related variability [15].

The findings of this study revealed that the RM system produced the least amount of apical debris, with a significant difference found between the RM and both WOG and PTN systems. Being a single reciprocating file system, the RM produced the lowest amount of AED, which can be attributed to its slender core combined with the newly modified spherical tip. Furthermore, the triangular cross-sectional design of blue heat-treated wire might explain this file's reduced amount of AED since previous studies have confirmed that the triangular cross section promotes the instrument's flexibility and facilitates debris removability [30]. Furthermore, a previous systematic review has indicated that the instrument design significantly influences producing apical debris more than the number of files [31].

Although the WOG and RM systems both shared the same kinematics of reverse reciprocation motion with 150 CCW > 30 CW angles, the RM extruded significantly less debris than the WOG system. This result can be attributed to the difference between WOG and RM in the cross-section design and taper. The taper of WOG being 7% at the apex with regressive nature resulting in a larger core size of a parallelogram cross section. The present study's results are consistent with previous studies, which concluded that core size and cross-sectional design features could be detrimental to the AED [17, 31].

In the present study, the PTN system has produced a significantly higher amount of debris than the RM file system. The PTN, with a rectangular cross section, off-centered rotary motion, and regressive–progressively tapered file as a multifile system, has all these features designed on M-wire alloy technology. Previous studies have suggested that the swaggering motion of PTN tend to allow debris to be removed coronally, which enhances it's cutting efficacy by allowing the file to cut dentin in larger envelop of motion [22, 32]. Alternatively, the minimally invasive design of the RM could be one reason in reducing the amount of AED in this study. Moreover, the difference in file's number, design, and metallurgy can also be considered as an influential factor.

Additionally, the X1 file has a square cross-sectional design to fortify its apical segment [14]. However, this design feature could have increased this file's screwing in effect with the resulting debris extrusion, [33] because the screwing-in effect may lead to accidental over instrumentation of the apical foramen, which can further increase the apical extrusion of debris beyond the canal [34].

Previous reports showed that the resultant pitch elongation of the HFEDM files due to their uncoiling characteristics might tend to extrude more apical debris [14, 18]. In the current investigation, the operator observed HFEDM files deforming during instrumentation in the present study, which led to an increase in the working length in 75% of samples tested in the HFEDM group. This observation can explain that debris extrusion may have occurred due to apical enlargement, while overinstrumentation of the apical foramen [35]. In the present study, the HFEDM system extruded more debris than the RM system; however, the difference was insignificant. These results correlate to the fact that the HFEDM OneFile shares a similar cross-sectional design at its coronal part to the RM file.

Regardless of the variations between the PTN and WOG systems, such as the number of files, motion, taper, and WOG file's controlled-memory feature, the current study found no significant difference between them. The two systems share the same cross-sectional design characteristic with an off-centered axis of rotation, which explains the outcome of this study. A recent study by Kharouf et al. [17] agreed with this result and compared PTN in full rotation and reciprocation with WOG, resulting in no significant difference between the tested files sharing a similar rectangular cross section. Another study by Eliasz et al. [36] revealed that PTN and WOG produced the same amount of AED. On the contrary, previous studies found that reciprocating instruments may cause more AED than full rotary instruments [37, 38]. However, the present study tested differently designed reciprocating systems, metallurgy, and sample characteristics.

In the current study, the HFEDM system extruded less debris than PTN and WOG with no significant difference, which is consistent with previous studies [18, 39]. Even though HFEDM has different kinematics, alloy, and file design than WOG and PTN, the HFEDM system shares a similar apical cross section (from D0 to D3), which can explain the absence of a significant difference in the amount of AED between these three tested files. Moreover, the lack of significant differences between WOG, PTN, and HFEDM is consistent with a previous randomized controlled trial in which there were no significant differences between these systems [40].

Although the present study's findings contradicted a study by Mustafa et al. [29]. who concluded that HFEDM produced significantly more debris than PTN In their research, they used severely curved mesiobuccal canals, which might accentuate the unwinding deformation that HFEDM wire undergoes due to stress.

A high standard deviation is common in AED studies, as encountered previously in Myers and Montgomery's original study [41]. The consistent presence of outliers in such studies may explain the nonnormal distribution of data in the present study. Moreover, the presence of outliers in these kinds of studies might be attributed to the inherent variability in the dentin microhardness of different samples.

The limitation of the method by Myers and Montgomery is that it only considers the quantitative aspect of extruded debris rather than its quality or bacterial virulence. Moreover, this method simulates neither pulpal nor periapical resistance, which might counter the pressure applied during shaping and cleaning. Thus, correlating the findings of the present laboratory with clinical practice requires further in vivo studies.

## 5. Conclusion

Within the limitations of this in vitro study, all tested groups produced varying amounts of AED. The WOG, PTN, and HFEDM caused a comparable quantity of AED; however, it can be argued that the RM system is a safer option during root canal treatment since it has produced less or equivalent amount of AED than the other tested systems.

## Figures and Tables

**Table 1:** The mean, standard deviation (SD), standard error (SE), median, minimum (Min), and maximum (Max) amounts of apically extruded debris by all tested groups in (mg).

Groups	Mean (mg)	SD	SE	Median	Min	Max	*P*-values
RM (I)	0.00015	0.00008	0.00002	0.00016	0.00004	0.00032	
WOG (II)	0.00025	0.00012	0.00003	0.00023	0.00010	0.00054	0.032
PTN (III)	0.00032	0.00018	0.00004	0.00029	0.00010	0.00076	
HFEDM (IV)	0.00023	0.00017	0.00004	0.00018	0.00008	0.00066	

*Note*. The significant level set at *P* < 0.05, the Kruskal–Willis test suggested that there was a significant difference between the tested groups, as the *P*-value was less than 0.05.

**Table 2:** Mann–Whitney test compares AED between every two groups.

Groups	Number of samples	Mean rank	Sum of ranks	Mann–Whitney *U* test	*P*-values
RM (I)	14	11.25	157.50	52.500	0.036
WOG (II)	14	17.75	248.50		
RM (I)	14	10.36	145.00	40.000	0.008
PTN (III)	14	18.64	261.00		
RM (I)	14	12.89	180.50	75.500	0.300
HFEDM (IV)	14	16.11	225.50		
WOG (II)	14	12.68	177.50	72.500	0.240
PTN (III)	14	16.32	228.50		
WOG (II)	14	15.89	222.50	78.500	0.369
HFEDM (IV)	14	13.11	183.50		
PTN (III)	14	17.07	239.00	62.000	0.098
HFEDM (IV)	14	11.93	167.00		

*Note*. The significant level was set at *P* < 0.05. Bold values illustrate a significant difference between tested groups.

## Data Availability

The data supporting the findings of the study are available from the corresponding author upon reasonable request.

## References

[1] Siqueira J. F. (2003). Microbial causes of endodontic flare-ups. *International Endodontic Journal*.

[2] De Deus G., Silva E. J. N. L., Souza E., Versiani M. A., Zuolo M. (2022). *Shaping for Cleaning the Root Canals: A Clinical-Based Strategy*.

[3] Çırakoglu N. Y., Özbay Y. (2021). Apically extruded debris associated with ProTaper next, ProTaper gold and TruNatomy systems: an in vitro study. *Journal of Dental Research, Dental Clinics, Dental Prospects*.

[4] Haridas K., Hariharan M., Singh P., Varughese A., Ravi A. B., Varma K. R. (2019). Effect of instrumentation techniques and kinematics on apical extrusion of debris: an in vitro study. *The Journal of Contemporary Dental Practice*.

[5] Tanalp J., Güngör T. (2014). Apical extrusion of debris: a literature review of an inherent occurrence during root canal treatment. *International Endodontic Journal*.

[6] Yared G. (2012). Canal preparation using one reciprocationg instrument without prior hand filing: a new concept. *International Dentistry*.

[7] Arias A., Peters O. A. (2022). Present status and future directions: canal shaping. *International Endodontic Journal*.

[8] Ruddle C. J. (2012). Canal preparation: single-file shaping technique. *Dentistry Today*.

[9] Bürklein S., Benten S., Schäfer E. (2014). Quantitative evaluation of apically extruded debris with different single-file systems: reciproc, F360 and OneShape versus MTWO. *International Endodontic Journal*.

[10] Tinoco J. M., De-Deus G., Tinoco E. M. B., Saavedra F., Fidel R. A. S., Sassone L. M. (2014). Apical extrusion of bacteria when using reciprocating single-file and rotary multifile instrumentation systems. *International Endodontic Journal*.

[11] Üstün Y., Çanakçi B. C., Dinçer A. N., Er O., Düzgün S. (2015). Evaluation of apically extruded debris associated with several NiTi systems. *International Endodontic Journal*.

[12] de Carvalho K. K. T., Petean I. B. F., Silva-Sousa A. C. (2022). Impact of several NiTi-thermally treated instrumentation systems on biomechanical preparation of curved root canals in extracted mandibular molars. *International Endodontic Journal*.

[13] Tanalp J. (2022). A critical analysis of research methods and experimental models to study apical extrusion of debris and irrigants. *International Endodontic Journal*.

[14] Capar I. D., Arslan H., Akcay M., Ertas H. (2014). An *in vitro* comparison of apically extruded debris and instrumentation times with protaper universal, protaper next, twisted file adaptive, and hyflex instruments. *Journal of Endodontics*.

[15] Koçak M. M., Çiçek E., Koçak S., Sağlam B. C., Furuncuoğlu F. (2016). Comparison of ProTaper next and HyFlex instruments on apical debris extrusion in curved canals. *International Endodontic Journal*.

[16] Schneider S. W. (1971). A comparison of canal preparations in straight and curved root canals. *Oral Surgery, Oral Medicine, Oral Pathology*.

[17] Kharouf N., Pedullà E., Nehme W. (2022). Apically extruded debris in curved root canals using a new reciprocating single-file shaping system. *Journal of Endodontics*.

[18] Azim A. A., Wang H. H., Tarrosh M., Azim K. A., Piasecki L. (2018). Comparison between single-file rotary systems: Part 1—efficiency, effectiveness, and adverse effects in endodontic retreatment. *Journal of Endodontics*.

[19] Karam M., Zogheib C., Khalil I. (2021). Shaping ability of WaveOne gold reciprocating file with and without glidepath in artificial S-shaped canals. *Giornale Italiano Di Endodonzia*.

[20] Dagna A., El Abed R., Hussain S. (2017). Comparison of apical extrusion of intracanal bacteria by various glide-path establishing systems: an in vitro study. *Restorative Dentistry & Endodontics*.

[21] Al-tayyar S. S., Shukri B. M. S. (2020). The amount of extruded debris: (an In-vitro com- parative study). *Journal of Oral and Dental Research*.

[22] Yılmaz K., Özyürek T. (2017). Apically extruded debris after retreatment procedure with reciproc, ProTaper next, and twisted file adaptive instruments. *Journal of Endodontics*.

[23] Pirani C., Iacono F., Generali L. (2016). HyFlex EDM: superficial features, metallurgical analysis, and fatigue resistance of innovative electro discharge machined NiTi rotary instruments. *International Endodontic Journal*.

[24] Çapar I. D., Arslan H. (2016). A review of instrumentation kinematics of engine-driven nickel–titanium instruments. *International Endodontic Journal*.

[25] De-Deus G., Brandão M. C., Barino B., Di Giorgi K., Fidel R. A. S., Luna A. S. (2010). Assessment of apically extruded debris produced by the single-file ProTaper F2 technique under reciprocating movement. *Oral Surgery, Oral Medicine, Oral Pathology, Oral Radiology, and Endodontology*.

[26] Hussein H. M., Al-zaka I. M. (2014). Evaluation of the amount of apically extruded debris using different root canal instrumentation systems. *Mustansiria Dental Journal*.

[27] Bürklein S., Schäfer E. (2012). Apically extruded debris with reciprocating single-file and full-sequence rotary instrumentation systems. *Journal of Endodontics*.

[28] Arias A., de la Macorra J. C., Hidalgo J. J., Azabal M. (2013). Predictive models of pain following root canal treatment: a prospective clinical study. *International Endodontic Journal*.

[29] Mustafa R., Al Omari T., Al-Nasrawi S., Al Fodeh R., Dkmak A., Haider J. (2021). Evaluating in vitro performance of novel Nickel-Titanium rotary system (TruNatomy) based on debris extrusion and preparation time from severely curved canals. *Journal of Endodontics*.

[30] Xu K., Wang J., Wang K., Gen N., Li J. (2018). Micro-computed tomographic evaluation of the effect of the final apical size prepared by rotary nickel–titanium files on the removal efficacy of hard-tissue debris. *Journal of International Medical Research*.

[31] Caviedes-Bucheli J., Castellanos F., Vasquez N., Ulate E., Munoz H. R. (2016). The influence of two reciprocating single-file and two rotary-file systems on the apical extrusion of debris and its biological relationship with symptomatic apical periodontitis. a systematic review and meta-analysis. *International Endodontic Journal*.

[32] keskin C., Sarıyılmaz E. (2018). Apically extruded debris and irrigants during root canal filling material removal using reciproc blue, WaveOne gold, R-Endo and ProTaper next systems. *Journal of Dental Research, Dental Clinics, Dental Prospects*.

[33] Ha J. H., Cheung G. S. P., Versluis A., Lee C. J., Kwak S. W., Kim H. C. (2015). Screw-in tendency of rotary nickel-titanium files due to design geometry. *International Endodontic Journal*.

[34] Ha J.-H., Kwak S. W., Kim S.-K., Kim H.-C. (2016). Screw-in forces during instrumentation by various file systems. *Restorative Dentistry & Endodontics*.

[35] Silva E. A. B., Guimarães L. S., Küchler E. C., Antunes L. A. A., Antunes L. S. (2017). Evaluation of effect of foraminal enlargement of necrotic teeth on postoperative symptoms: a systematic review and meta-analysis. *Journal of Endodontics*.

[36] Eliasz W., Czarnecka B., Surdacka A. (2021). Apical extrusion of debris during root canal preparation with protaper next, waveone gold and twisted files. *Materials*.

[37] Surakanti J. R., Venkata R. C. P., Vemisetty H. K., Dandolu R. K., Jaya N. K. M., Thota S. (2014). Comparative evaluation of apically extruded debris during root canal preparation using ProTaperTM, HyflexTM and WaveoneTM rotary systems. *Journal of Conservative Dentistry*.

[38] Pawar A. M., Pawar M. G., Metzger Z., Kokate S. R. (2015). The self-adjusting file instrumentation results in less debris extrusion apically when compared to WaveOne and ProTaper NEXT. *Journal of Conservative Dentistry*.

[39] Adnan M., Gharrawi H. (2021). Evaluation of the apically extruded debris during canal preparation by WaveOne gold, Hyflex EDM and XP-endo shaper, Anin. *Sulaimani Dental Journal*.

[40] Çanakçi B. C., Er Ö., Genç Şen Ö., Süt N. (2021). The effect of two rotary and two reciprocating NiTi systems on postoperative pain after root canal retreatment on single-rooted incisor teeth: a randomized controlled trial. *International Endodontic Journal*.

[41] Myers G. L., Montgomery S. (1991). A comparison of weights of debris extruded apically by conventional filing and canal master techniques. *Journal of Endodontics*.

